# Systemic Immune–Inflammation Index, Thrombotic Status, and Mortality in Patients with Cancer: A Matched Cohort Study

**DOI:** 10.3390/diseases14080285

**Published:** 2026-08-09

**Authors:** Bozhidar Krastev, Natalia Spasova, Georgi Dimitrov, Elena Kinova, Assen Goudev

**Affiliations:** 1 Department of Emergency Medicine, Medical University of Sofia, 1527 Sofia, Bulgaria; spasova.natalia@gmail.com (N.S.); kinova.e@abv.bg (E.K.); agoudev@abv.bg (A.G.); 2 Clinic of Cardiology, University Hospital “Tsaritsa Yoanna-ISUL”, 1527 Sofia, Bulgaria; 3 Department of Medical Oncology, Medical University of Sofia, University Hospital “Tsaritsa Yoanna-ISUL”, 1527 Sofia, Bulgaria; g.dimitrov.md@gmail.com

**Keywords:** cancer-associated thrombosis, systemic immune–inflammation index, all-cause mortality, solid cancers, matched cohort

## Abstract

**Background:** Cancer-associated thrombosis is a clinically important complication of malignancy. The systemic immune–inflammation index (SII), calculated from platelet, neutrophil, and lymphocyte counts, may reflect the inflammatory background of cancer, but its value in matched heterogeneous cancer cohorts remains unclear. **Objective:** To evaluate whether SII is associated with thrombotic status and recorded all-cause mortality in patients with solid cancers. **Methods:** This retrospective matched cohort study was derived from 2020 consecutive adult patients hospitalized with solid malignancies between September 2023 and December 2025 at the Oncology Clinic of University Hospital “Tsaritsa Yoanna—ISUL”, Sofia, Bulgaria. Patients with documented thrombosis were matched 1:2 to non-thrombotic controls using a nearest propensity-score approach, with preference for exact matching on sex and cancer type. The final cohort included 110 thrombotic patients and 220 matched controls. SII was analyzed in relation to thrombotic status and recorded all-cause mortality using logistic regression. **Results:** Thrombotic patients and controls were well balanced for matching variables. Mortality was similar between groups: 29.1% versus 28.2%. SII did not differ significantly between thrombotic patients and controls: 772 (516–1471) versus 783 (501–1292), *p* = 0.655, and showed poor discrimination for thrombotic status, with an AUC of 0.515. In the overall matched cohort, higher SII was associated with recorded all-cause mortality after adjustment for thrombotic status, age, sex, cancer type, stage, and metastatic disease: OR 1.31 per 1000-unit increase, 95% CI 1.05–1.64, *p* = 0.016. The association was numerically stronger among thrombotic patients, but interaction testing was not statistically significant. **Conclusions:** SII was not associated with thrombotic status in this matched cancer cohort. Higher SII was associated with recorded all-cause mortality, suggesting that SII may better reflect systemic inflammatory burden than cancer-associated thrombosis itself.

## 1. Introduction

Cancer remains a major cause of morbidity and mortality worldwide, alongside cardiovascular disease [1]. Among the complications seen in patients with malignancy, cancer-associated thrombosis is particularly important in daily clinical practice.

The relationship between cancer and thrombosis has been recognized since the second half of the 19th century, when Armand Trousseau described the association between thrombotic events and occult malignancy, later known as Trousseau’s syndrome [2].

Subsequent clinical observations showed that patients with advanced cancer may develop thrombocytosis [3]. Later studies further clarified that platelets are not only involved in clot formation, but can also participate in tumor growth, angiogenesis, and metastatic spread, supporting their role as potential markers of tumor aggressiveness [4].

Cancer-associated thrombosis develops in a complex prothrombotic setting. Tumor-related hypercoagulability, anticancer treatment, and patient-related factors such as comorbidities and genetic predisposition may all contribute to thrombotic risk.

Thromboembolic complications are considered one of the leading causes of death in patients with cancer after progression of the underlying malignancy. Patients with cancer have a markedly higher incidence of deep vein thrombosis, and malignancy may be found in a substantial proportion of patients presenting with deep vein thrombosis [5,6].

Thrombosis at unusual sites, including mesenteric vein thrombosis, Budd-Chiari syndrome, and extrahepatic portal vein obstruction, may also be an initial manifestation of an underlying malignant disease [7].

In clinical practice, this means that a thromboembolic event may sometimes be the first reason to search actively for an occult cancer.

The systemic immune–inflammation index (SII), calculated as platelet count × neutrophil count/lymphocyte count, is a simple blood count-derived biomarker that integrates inflammatory and immune responses. Higher SII values reflect the combination of neutrophilia and thrombocytosis, together with relative lymphopenia, a pattern associated with systemic inflammation, tumor progression, and adverse clinical outcomes in several malignancies [8].

In this setting, simple blood count-derived inflammatory indices may provide additional information, but their value in heterogeneous cancer populations remains uncertain. This retrospective study aims to evaluate whether SII is associated with thrombotic status and recorded all-cause mortality in a matched cohort of patients harboring a solid malignancy.

## 2. Materials and Methods

### 2.1. Study Design and Population

This retrospective observational matched cohort study was based on a source cohort of 2020 consecutive adult patients hospitalized with a solid malignancy between September 2023 and December 2025 at the Oncology Clinic of University Hospital “Tsaritsa Yoanna—ISUL”, Sofia, Bulgaria.

Patients were grouped by tumor type as respiratory, gastrointestinal, genitourinary, breast, or other non-hematologic cancers. Demographic data, cancer-related characteristics, cardiovascular comorbidities, cardiovascular risk factors, laboratory results, and outcome status were extracted from electronic medical records. Cancer stage and metastatic disease status were recorded.

Available information on oncological treatment and antithrombotic therapy was also extracted from the medical records. Because these treatment data were not available for the matched controls, they were analyzed descriptively only among patients with thrombosis.

For the present analysis, patients with documented thrombotic events were selected from the source cohort. The thrombotic cohort included both patients with a documented thrombotic event before the index hospitalization and patients who developed thrombosis during follow-up after baseline assessment. Thrombotic events included deep vein thrombosis, pulmonary embolism, and thrombosis at other venous sites. The latter category comprised a heterogeneous group of less common venous thromboses, including splanchnic thrombosis, jugular vein thrombosis, upper-extremity vein thrombosis, and other unusual-site venous thromboses. Each thrombotic patient was matched with non-thrombotic controls from the same cohort according to age, sex, cancer type, cancer stage, and metastatic disease status.

The final matched cohort included 110 patients with thrombosis and 220 matched non-thrombotic controls.

### 2.2. Matching Procedure

Matching was performed to reduce baseline differences between patients with and without thrombosis. For each patient with documented thrombosis, two non-thrombotic controls were selected from the same source cohort whenever suitable controls were available. Patients with any recorded pulmonary embolism, deep vein thrombosis or other-site venous thrombosis were not eligible to serve as controls. Matching was performed without replacement using a nearest propensity-score approach, with preference for exact matching on sex and cancer type. After matching, the balance between groups was assessed for the main demographic, oncological, cardiovascular, and lifestyle-related variables using standardized mean differences.

### 2.3. Definition of Thrombotic Status

Thrombotic status was defined by the presence or absence of a documented thrombotic event in the available medical records up to the time of data extraction. Patients with documented thrombosis formed the thrombotic group, whereas matched patients without any recorded thrombotic event served as controls. Thrombotic events were classified as described above.

### 2.4. Laboratory Parameters and Inflammatory Indices

Baseline laboratory parameters were obtained from hospital records. The analyzed variables included hemoglobin, leukocytes, neutrophils, lymphocytes, monocytes, platelets, and creatinine.

Baseline laboratory parameters were obtained during the first hospitalization before the initiation of treatment.

Inflammatory indices were calculated from complete blood count parameters as follows:

**NLR = neutrophil count/lymphocyte count**

**PLR = platelet count/lymphocyte count**

**SII = platelet count × neutrophil count/lymphocyte count**

Additional inflammatory indices, including LMR and MLR, were evaluated in supplementary analyses.

### 2.5. Outcome Definition

The primary outcome was recorded all-cause mortality at the time of data extraction. Mortality status was ascertained through the National Health Insurance Fund database and hospital electronic medical records.

Because exact dates of death and complete time-to-event information were not available for all patients, mortality was analyzed as a binary outcome using logistic regression. The results should therefore be interpreted as associations with recorded mortality, not as time-to-event risk estimates.

### 2.6. Time to Thrombotic Event

Of the 110 patients with thrombosis, 33 had experienced the thrombotic event before baseline assessment. Exact time from baseline assessment to the documented thrombotic event was therefore available for the remaining 77 patients. In these patients, the interval was calculated in days and summarized descriptively using the median, interquartile range, range, and predefined time categories (≤7, ≤30, ≤90, ≤180, and ≤365 days). Owing to the absence of corresponding follow-up and censoring data for the matched controls, comparative time-to-event analyses were not performed.

### 2.7. Statistical Analysis

Statistical analyses were performed using SPSS software, version 23.0 (IBM Corp., Armonk, NY, USA).

Continuous variables were summarized as median and interquartile range because most laboratory and inflammatory variables were not normally distributed. Categorical variables were presented as counts and percentages.

Continuous variables were compared between thrombotic patients and matched controls using the Mann–Whitney U test. Categorical variables were compared using the chi-square test or Fisher’s exact test, as appropriate.

The association between SII and thrombotic status was assessed by comparing SII values between thrombotic patients and matched controls and by logistic regression analysis. The discriminatory ability of SII for thrombotic status was evaluated using the area under the receiver operating characteristic curve.

Cancer-specific subgroup analyses were performed by comparing SII between patients with thrombosis and matched controls within each predefined cancer category using the Mann–Whitney U test.

The association between inflammatory indices and recorded all-cause mortality was assessed separately in thrombotic patients and matched controls. Multivariable binary logistic regression models were used to evaluate the independent association between SII and all-cause mortality. Because mortality was analyzed as the primary outcome within the matched analytical cohort, rather than as the matching outcome itself, the models were adjusted for the matching variables.

In the overall matched cohort, the main multivariable model included thrombotic status, age, sex, cancer type, cancer stage, metastatic disease, and SII. In subgroup analyses restricted to thrombotic patients and matched controls, models included age, sex, cancer stage, metastatic disease, and SII. SII was entered as a continuous variable per 1000-unit increase.

Model discrimination was assessed using the area under the receiver operating characteristic curve. Calibration was evaluated using the Hosmer–Lemeshow goodness-of-fit test. The incremental value of SII was assessed by likelihood ratio tests comparing nested models with and without SII. An interaction analysis was performed to explore whether the association between SII and mortality differed according to thrombotic status.

A sensitivity analysis was performed by additionally adjusting the main model for hemoglobin and creatinine. Additional inflammatory and hematological markers were evaluated in supplementary analyses.

All statistical tests were two-sided, and a *p* value <0.05 was considered statistically significant.

### 2.8. Ethics

The study was conducted in accordance with the Declaration of Helsinki and was approved by the institutional ethics committee of University Hospital “Tsaritsa Yoanna—ISUL”, Sofia, Bulgaria. Due to the retrospective nature of the study, the requirement for informed consent was waived.

## 3. Results

### 3.1. Study Population and Baseline Characteristics

The matched cohort included 330 patients with cancer, comprising 110 patients with thrombosis and 220 matched non-thrombotic controls. The two groups were well balanced for all predefined matching variables (Table 1). The improvement in baseline balance after matching was further supported by standardized mean differences, as shown in Supplementary Table S1.

Median age was similar in thrombotic patients and matched controls: 71.0 years [64.3–75.0] versus 71.0 years [63.8–76.0], respectively (*p* = 0.919). The proportion of male patients was identical in both groups: 52.7% in thrombotic patients and 52.7% in matched controls (*p* = 1.000).

The distribution of cancer types was fully balanced between the two groups (*p* = 1.000). Gastrointestinal malignancies were the most frequent cancer type, observed in 41.8% of patients in both groups, followed by genitourinary, breast, respiratory, and other cancers.

Cancer stage distribution was also comparable between thrombotic patients and matched controls (*p* = 0.990). Stage IV disease was present in 40.0% of thrombotic patients and 39.5% of matched controls. Metastatic disease was observed in 31.8% and 30.0% of patients, respectively (*p* = 0.800).

All-cause mortality was similar in the two groups, occurring in 32 thrombotic patients (29.1%) and 62 matched controls (28.2%) (*p* = 0.897).

Overall, these findings indicate that the matching procedure achieved good balance between thrombotic patients and non-thrombotic controls with respect to demographic and oncological characteristics. In this matched cohort, thrombotic status itself was not associated with higher all-cause mortality.

### 3.2. Time from Baseline Assessment to Thrombotic Event

Exact time from baseline assessment to the documented thrombotic event was available for 77 of the 110 patients with thrombosis (70.0%). The median interval was 104 days (IQR 30–286), with a range of 0–1312 days. Twenty thrombotic events (26.0%) occurred within 30 days, 32 (41.6%) within 90 days, 51 (66.2%) within 180 days, and 60 (77.9%) within one year. Five thrombotic events (6.5%) were documented on the day of the baseline assessment. Detailed descriptive data are presented in Supplementary Table S2.

The distribution of thrombotic event types is presented in Supplementary Table S2. Pulmonary embolism was the most frequent thrombotic event, occurring in 47 patients (42.7%), followed by deep vein thrombosis in 42 patients (38.2%). Thrombosis at other venous sites was documented in 21 patients (19.1%) and included upper-extremity, portal, jugular, renal, and other venous thromboses.

**Figure 1:**
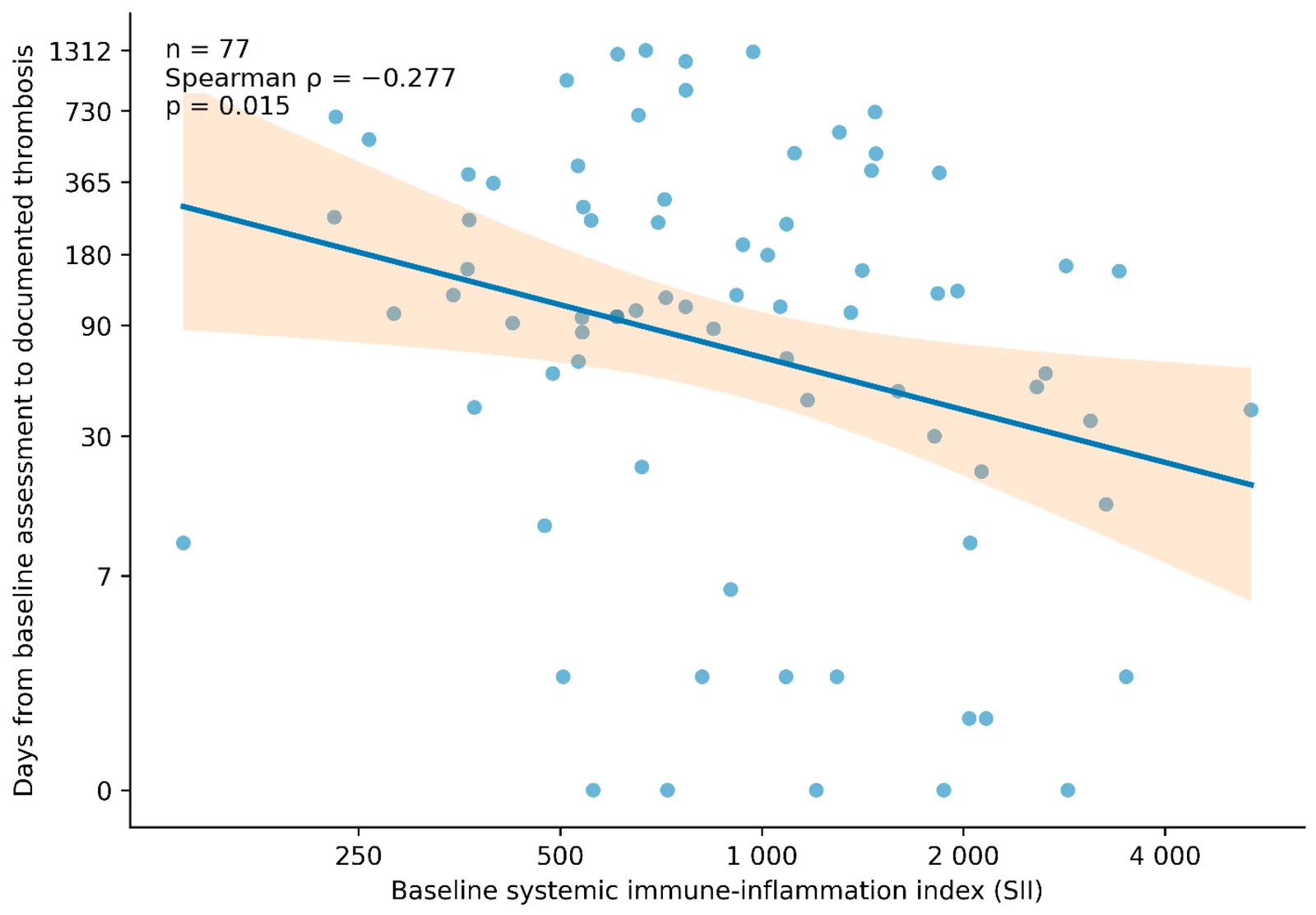
Association between baseline systemic immune–inflammation index and time to documented thrombosis.

Higher baseline SII was weakly associated with a shorter interval to the documented thrombotic event (Spearman ρ = −0.277, *p* = 0.015). This relationship is illustrated in Figure 1, which shows the association between baseline SII and time to thrombosis among the 77 patients with available timing data. These findings should be interpreted as descriptive information on event timing rather than as estimates of thrombotic risk over time.

Individual points represent the 77 patients with an available exact interval from baseline assessment to the thrombotic event. The solid line and shaded area represent the fitted linear trend and its 95% confidence interval based on log2-transformed SII and log-transformed time. Higher baseline SII was weakly associated with a shorter interval to documented thrombosis (Spearman ρ = −0.277, *p* = 0.015). This case-only exploratory analysis describes event timing and should not be interpreted as an estimate of incident thrombotic risk. Because follow-up and censoring data were unavailable for the matched controls, comparative time-to-event analyses could not be performed. Therefore, this figure is descriptive and should not be interpreted as a survival or time-to-event analysis.

Cardiovascular comorbidities and conventional risk factors were comparable between thrombotic patients and matched non-thrombotic controls (Table 2).

Coronary artery disease was present in 5 thrombotic patients (4.5%) and 8 matched controls (3.6%) (*p* = 0.766). Previous myocardial infarction was observed in 4.5% versus 4.1% of patients, respectively (*p* = 1.000), while previous stroke was present in 6.4% of thrombotic patients and 5.0% of matched controls (*p* = 0.614).

The prevalence of diabetes mellitus, hypertension, obesity, atrial fibrillation, dyslipidemia, and heart failure did not differ significantly between groups. Hypertension was the most frequent cardiovascular risk factor, observed in 68.2% of thrombotic patients and 72.7% of matched controls (*p* = 0.439). Diabetes mellitus was present in 20.0% and 22.3%, respectively (*p* = 0.672), and obesity in 24.5% and 28.2%, respectively (*p* = 0.513).

Lifestyle-related risk factors were also similar. Smoking was reported in 19.1% of thrombotic patients and 19.5% of matched controls (*p* = 1.000), while alcohol use was present in 12.7% of patients in both groups (*p* = 1.000).

Overall, there were no statistically significant differences in cardiovascular comorbidities or risk factors between thrombotic patients and matched controls, supporting good clinical balance between the two groups beyond the predefined matching variables.

Baseline laboratory parameters and inflammatory indices were similar between thrombotic patients and matched non-thrombotic controls (Table 3).

Median hemoglobin levels were comparable between the two groups: 133.5 g/L [124.0–142.8] in thrombotic patients versus 133.0 g/L [121.0–143.0] in matched controls (*p* = 0.794). Leukocyte counts were also similar: 7.54 × 10^9^/L [5.90–9.03] versus 7.50 × 10^9^/L [5.75–9.81], respectively (*p* = 0.849).

No significant differences were observed in neutrophil, lymphocyte, monocyte, or platelet counts. Median neutrophil count was 4.90 × 10^9^/L [3.92–6.63] in thrombotic patients and 4.79 × 10^9^/L [3.70–6.70] in matched controls (*p* = 0.694). Platelet counts were also comparable: 246.5 × 10^9^/L [201.5–317.8] versus 255.5 × 10^9^/L [200.5–321.5], respectively (*p* = 1.000). Creatinine levels did not differ significantly between groups (*p* = 0.457).

Inflammatory indices were also similar between thrombotic patients and matched controls. Median NLR was 3.17 [2.14–5.16] in thrombotic patients and 3.07 [2.11–4.85] in matched controls (*p* = 0.543). Median PLR was numerically higher in thrombotic patients, but the difference was not statistically significant: 175 [115–268] versus 154 [119–224] (*p* = 0.559).

Median SII was 772 [516–1471] in thrombotic patients and 783 [501–1292] in matched controls (*p* = 0.655). In univariable logistic regression analysis, SII was not associated with documented thrombotic status (OR 0.95 per 1000-unit increase, 95% CI 0.83–1.08, *p* = 0.425). Consistent with this finding, the discriminatory performance of SII was poor (AUC 0.515, 95% CI 0.449–0.581). Thus, SII did not differ significantly between patients with thrombosis and matched non-thrombotic controls.

Overall, these findings indicate that thrombotic patients and matched controls had comparable baseline hematological profiles and systemic inflammatory indices. In this matched cohort, SII, NLR, and PLR were not associated with thrombotic status.

### 3.3. Cancer-Specific Analyses

Cancer-specific analyses showed no statistically significant differences in SII between patients with thrombosis and matched controls within the respiratory, gastrointestinal, genitourinary, breast, or other cancer categories. The corresponding *p* values were 0.922, 0.650, 0.649, 0.849, and 0.898, respectively (Supplementary Table S3).

### 3.4. Multivariable Logistic Regression Models for All-Cause Mortality

Multivariable logistic regression was performed to evaluate the association between SII and all-cause mortality (Table 4).

In the overall matched cohort, SII was associated with recorded all-cause mortality after adjustment for thrombotic status, age, sex, cancer type, cancer stage, and metastatic disease. Each 1000-unit increase in SII was associated with higher odds of mortality: OR 1.31, 95% CI 1.05–1.64, *p* = 0.016. The model showed good discrimination, with an AUC of 0.779.

When the analysis was repeated separately in the two groups, the association was numerically more pronounced among patients with thrombosis: OR 2.10, 95% CI 1.24–3.57, *p* = 0.006; AUC = 0.818. In matched controls, the association did not reach statistical significance: OR 1.18, 95% CI 0.95–1.45, *p* = 0.131; AUC = 0.779.

Although the association was numerically stronger in thrombotic patients, formal interaction testing did not confirm a statistically significant difference between groups. Therefore, these subgroup findings should be interpreted as exploratory.

### 3.5. Model Performance, Incremental Value, and Sensitivity Analyses

The multivariable mortality models showed good discrimination and adequate calibration. The overall model had an AUC of 0.779 and adequate calibration by the Hosmer–Lemeshow test (*p* = 0.462). Discrimination was highest among thrombotic patients (AUC = 0.818; Hosmer–Lemeshow *p* = 0.244) and remained acceptable among matched controls (AUC = 0.779; *p* = 0.244) (Supplementary Table S4).

Adding SII significantly improved model fit in the overall matched cohort (LR χ^2^ = 9.81, *p* = 0.0017) and in thrombotic patients (LR χ^2^ = 8.94, *p* = 0.0028), with a smaller but statistically significant effect among matched controls (LR χ^2^ = 5.11, *p* = 0.024). The SII × thrombotic status interaction was not statistically significant (LR χ^2^ = 3.26, *p* = 0.071) (Supplementary Table S5).

In sensitivity analysis, SII remained associated with mortality after additional adjustment for hemoglobin and creatinine: OR 1.29, 95% CI 1.02–1.63, *p* = 0.031 (Supplementary Table S6). The nested likelihood ratio test shown in this sensitivity model reflects the joint addition of SII, hemoglobin, and creatinine, and should therefore not be interpreted as the incremental value of SII alone. Additional inflammatory and hematological marker models and SII cut-off analyses are shown in Supplementary Tables S7 and S8.

In an exploratory analysis restricted to thrombotic patients, mortality differed across oncological treatment categories, with the highest rates observed among patients receiving combined therapy or chemotherapy alone (Supplementary Table S9).

The distribution of primary tumor sites among patients with thrombosis is presented in Supplementary Table S10. Organ-specific tumor location and treatment data were available only for the thrombotic subgroup and are therefore presented descriptively without comparison with matched controls.

Information on antithrombotic treatment was available for 95 of the 110 patients with thrombosis. DOACs were the most frequently recorded treatment, used in 50 patients (45.5% of the thrombotic cohort), followed by low-molecular-weight heparin in 32 patients (29.1%), vitamin K antagonists in 9 patients (8.2%), and antiplatelet therapy in 4 patients (3.6%). Treatment information was unavailable for 15 patients. The full distribution is shown in Supplementary Table S10.

## 4. Discussion

Cancer-associated thrombosis remains one of the clinically important complications of malignancy and is associated with substantial morbidity and mortality [9,10,11,12]. In this matched cohort, SII did not differ between patients with documented thrombosis and matched non-thrombotic controls. This finding suggests that, in a heterogeneous cancer population balanced for age, sex, cancer type, stage, and metastatic status, SII has limited value as a standalone marker for identifying thrombotic status.

This result is biologically plausible. Cancer-associated VTE reflects several overlapping processes, including tumor-driven hypercoagulability, platelet activation, leukocyte involvement, endothelial dysfunction, treatment exposure, and patient-related factors [13]. SII captures only part of this complex background through platelet, neutrophil, and lymphocyte counts. Therefore, it may reflect systemic inflammatory and immune imbalance, but it cannot be expected to identify thrombosis independently of tumor burden and other clinical determinants.

Platelets are central to the connection between cancer, inflammation, and thrombosis. They interact with leukocytes, endothelial cells, and tumor cells, and may contribute to angiogenesis, immune escape, tumor cell survival, and metastatic spread [14]. Because SII combines platelet count with neutrophil predominance and relative lymphopenia, it has a reasonable biological basis as an inflammatory index in cancer. However, biological plausibility does not necessarily translate into diagnostic specificity for VTE.

The thrombotic cohort included patients with deep vein thrombosis, pulmonary embolism, and thrombosis at other venous sites. This reflects the heterogeneous clinical spectrum of cancer-associated venous thromboembolism encountered in routine oncology practice.

An additional consideration is that 33 patients (30.0% of the thrombotic cohort) had experienced thrombosis before the baseline assessment. These patients may already have been receiving anticoagulant therapy when SII was measured. Anticoagulation could potentially modify the underlying thrombo-inflammatory state; however, the timing, duration, and continuity of treatment in relation to blood sampling were not consistently available. Therefore, its possible effect on SII could not be assessed directly.

The prognostic value of SII in cancer has been supported by previous meta-analyses across different solid tumors, where higher SII was associated with worse survival and adverse cancer-related outcomes [15,16]. These studies mainly evaluated prognosis rather than thrombotic diagnosis. Our findings are consistent with this distinction: SII was not useful for separating thrombotic from non-thrombotic patients, but it was associated with recorded all-cause mortality.

Evidence from non-small cell lung cancer patients treated with immune checkpoint inhibitors has also shown that higher SII may be associated with poorer clinical outcomes, including shorter overall and progression-free survival [17]. Such data support the role of SII as an accessible blood count-derived marker associated with adverse clinical outcomes.

Several biomarkers have been studied for VTE prediction in cancer, including D-dimer, soluble P-selectin, platelet count, leukocyte count, and inflammatory markers [18,19]. Their clinical usefulness is generally greatest when they are interpreted together with tumor type, stage, metastatic disease, treatment characteristics, and established clinical risk models. Our results support a similar interpretation for SII [20].

Studies that specifically examined SII and VTE are still limited. Some retrospective data in lung cancer have suggested a diagnostic association between SII and VTE [21]. Another study in NSCLC patients receiving immune checkpoint inhibitors reported that high SII was independently associated with VTE, with a proposed cut-off of approximately 847.5 [22]. These findings are clinically relevant, but they come from selected tumor- and treatment-specific cohorts. They may not be directly applicable to a matched cohort including different cancer types and clinical settings.

This difference is important because previous studies of SII and VTE have often focused on single tumor sites, including lung or gastrointestinal cancers [23]. In a mixed oncology cohort, the inflammatory signal carried by one blood count-derived index may be diluted by differences in tumor biology, treatment exposure, infection, comorbidities, stage, and metastatic burden. To provide additional clinical context, the distribution of individual primary tumor sites within the thrombotic cohort is presented in Supplementary Table S10. However, organ-specific comparisons between thrombotic patients and matched controls were not possible because these data were unavailable for the control cohort. Matching for several of these factors may further reduce between-group differences in SII. Thus, the absence of a significant SII difference between thrombotic and non-thrombotic patients does not contradict the inflammatory basis of cancer-associated thrombosis. Rather, it indicates that SII is not specific enough to identify thrombosis once major oncological determinants are balanced.

The cancer-specific subgroup analyses were consistent with the overall findings, as SII did not differ significantly between patients with thrombosis and matched controls within any of the predefined cancer categories. However, these analyses should be interpreted cautiously because several cancer-specific subgroups contained relatively small numbers of thrombotic patients.

The mortality findings point in a different direction. SII remained associated with recorded all-cause mortality in the overall matched cohort, while the association was numerically more pronounced among thrombotic patients. However, the SII × thrombotic status interaction did not reach statistical significance. Therefore, these subgroup findings should be interpreted cautiously and should not be considered definitive evidence that the mortality-associated effect of SII differs according to thrombotic status.

Other blood count-derived indices also showed associations with recorded mortality in exploratory adjusted analyses. Higher NLR was associated with higher mortality, whereas higher LMR was inversely associated with mortality. PLR showed a nonsignificant trend toward higher risk.

These supplementary findings indicate that SII should not be interpreted as a unique inflammatory marker, but rather as one component of a broader blood count-derived inflammatory profile associated with recorded mortality.

Together, these findings support the broader concept that neutrophil predominance, platelet activation, and relative lymphopenia may reflect a more adverse clinical state in cancer patients. Similar observations have been reported in colorectal cancer with peritoneal carcinomatosis, where preoperative NLR, PLR, and MPV were associated with complications and poorer survival [24]. The clinical meaning of these markers, however, depends strongly on the endpoint being studied.

The sensitivity and model performance analyses support the main interpretation. The mortality model showed good discrimination and adequate calibration, and SII remained associated with mortality after additional adjustment for hemoglobin and creatinine. This suggests that the observed association was not explained only by anemia or renal dysfunction. Still, these findings should be viewed as prognostic associations with recorded mortality, not as time-to-event survival estimates.

Overall, our results suggest that SII is better interpreted as a marker of systemic inflammatory burden associated with recorded mortality than as an independent diagnostic marker for cancer-associated VTE. Prospective studies with standardized follow-up and serial inflammatory measurements are needed to determine whether SII can improve risk stratification in this setting.

The strengths of this study include the matched cohort design, the inclusion of a heterogeneous real-world oncology population, adjustment for major oncological determinants of outcome, and evaluation of SII both as a marker of thrombotic status and as an inflammatory index associated with recorded mortality.

This study has several limitations. First, its retrospective, single-center design limits causal inference and generalizability, and the observed associations should therefore be interpreted as hypothesis-generating. Although matching was performed for key clinical variables including age, sex, cancer type, stage, and metastatic status, residual and unmeasured confounding cannot be excluded. In addition, matching across broad oncological categories may have attenuated potential differences in SII between thrombotic and non-thrombotic patients.

Second, SII was derived from a single baseline blood sample, without longitudinal measurements. Consequently, temporal variability and dynamic changes in systemic inflammation could not be assessed, and the analysis reflects associations with documented thrombotic status rather than the predictive performance of SII for future thrombotic events. Furthermore, thrombotic status was determined from medical records, introducing the possibility of misclassification or under-detection of asymptomatic or unrecorded events.

Third, mortality was analyzed as a binary endpoint due to incomplete follow-up data and missing exact dates of death in a subset of patients. Therefore, results do not represent time-to-event survival analyses and should be interpreted as associations with recorded all-cause mortality. Cause-specific mortality was not available, preventing differentiation between cancer-related, thrombotic, cardiovascular, or treatment-related deaths. Multivariable logistic regression adjusted for matching variables was used to mitigate confounding, but the absence of survival modeling remains a key limitation.

Finally, treatment information was incomplete and was available only for the thrombotic subgroup. Therefore, oncological and antithrombotic therapies could be described but not compared between the matched cohorts. Detailed data on thromboprophylaxis, corticosteroid exposure, treatment timing, dose intensity, active infection, and performance status were not systematically available. The heterogeneity of the cancer population may have further diluted tumor-specific effects. Individual primary tumor site was available only for patients with thrombosis and not for the matched controls. Therefore, organ-specific comparisons between the matched cohorts could not be performed. The relatively small thrombotic subgroup also limits the robustness of the exploratory analyses.

## 5. Conclusions

In this matched cohort of patients with solid malignancies, SII did not differ between patients with cancer-associated thrombosis and matched non-thrombotic controls. In contrast, higher SII was associated with recorded all-cause mortality in the overall cohort. The association was numerically more pronounced among thrombotic patients, but formal interaction testing did not confirm a statistically significant difference between groups. These findings suggest that SII is more likely to reflect systemic inflammatory burden and mortality-associated risk than to serve as a marker of cancer-associated thrombosis itself.

## Figures and Tables

**Table 1:** Matching variables and clinical outcome.

Variable	Thrombotic Patients (n = 110)	Matched Controls (n = 220)	*p* Value
Age, years	71.0 [64.3–75.0]	71.0 [63.8–76.0]	0.919
Male sex	58/110 (52.7)	116/220 (52.7)	1.000
Cancer type			1.000
Respiratory	16/110 (14.5)	32/220 (14.5)	NE
Gastrointestinal	46/110 (41.8)	92/220 (41.8)	NE
Genitourinary	22/110 (20.0)	44/220 (20.0)	NE
Breast	17/110 (15.5)	34/220 (15.5)	NE
Other	9/110 (8.2)	18/220 (8.2)	NE
Cancer stage			0.990
Stage I	17/110 (15.5)	37/220 (16.8)	NE
Stage II	24/110 (21.8)	48/220 (21.8)	NE
Stage III	25/110 (22.7)	48/220 (21.8)	NE
Stage IV	44/110 (40.0)	87/220 (39.5)	NE
Metastatic disease	35/110 (31.8)	66/220 (30.0)	0.800
All-cause mortality	32/110 (29.1)	62/220 (28.2)	0.897

Values are presented as median [interquartile range] or n/N (%).

**Table 2:** Cardiovascular comorbidities and risk factors.

Variable	Thrombotic Patients (n = 110)	Matched Controls (n = 220)	*p* Value
Coronary artery disease	5/110 (4.5)	8/220 (3.6)	0.766
Previous myocardial infarction	5/110 (4.5)	9/220 (4.1)	1.000
Previous stroke	7/110 (6.4)	11/220 (5.0)	0.614
Diabetes mellitus	22/110 (20.0)	49/220 (22.3)	0.672
Hypertension	75/110 (68.2)	160/220 (72.7)	0.439
Obesity	27/110 (24.5)	62/220 (28.2)	0.513
Atrial fibrillation	10/110 (9.1)	24/220 (10.9)	0.703
Dyslipidemia	12/110 (10.9)	16/220 (7.3)	0.297
Heart failure	15/110 (13.6)	27/220 (12.3)	0.729
Smoking	21/110 (19.1)	43/220 (19.5)	1.000
Alcohol use	14/110 (12.7)	28/220 (12.7)	1.000

Values are presented as n/N (%).

**Table 3:** Laboratory parameters and inflammatory indices.

Variable	Thrombotic Patients (n = 110)	Matched Controls (n = 220)	*p* Value
Hemoglobin, g/L	133.5 [124.0–142.8]	133.0 [121.0–143.0]	0.794
Leukocytes, ×10^9^/L	7.54 [5.90–9.03]	7.50 [5.75–9.81]	0.849
Neutrophils, ×10^9^/L	4.90 [3.92–6.63]	4.79 [3.70–6.70]	0.694
Lymphocytes, ×10^9^/L	1.50 [1.12–1.91]	1.60 [1.20–2.01]	0.343
Monocytes, ×10^9^/L	0.41 [0.31–0.51]	0.41 [0.30–0.58]	0.591
Platelets, ×10^9^/L	246.5 [201.5–317.8]	255.5 [200.5–321.5]	1.000
Creatinine, µmol/L	72.0 [64.0–93.5]	72.0 [63.0–87.0]	0.457
NLR	3.17 [2.14–5.16]	3.07 [2.11–4.85]	0.543
PLR	175 [115–268]	154 [119–224]	0.559
SII	772 [516–1471]	783 [501–1292]	0.655

Values are presented as median [interquartile range]. Abbreviations: NLR, neutrophil-to-lymphocyte ratio; PLR, platelet-to-lymphocyte ratio; SII, systemic immune–inflammation index.

**Table 4:** Multivariable logistic regression models for all-cause mortality.

Population	Model Covariates	OR for SII	95% CI	*p* Value	AUC
Overall matched cohort, n = 330	Thrombotic status, age, sex, cancer type, stage, metastatic disease	1.31	1.05–1.64	0.016	0.779
Thrombotic patients, n = 110	Age, sex, stage, metastatic disease	2.10	1.24–3.57	0.006	0.818
Matched controls, n = 220	Age, sex, stage, metastatic disease	1.18	0.95–1.45	0.131	0.779

Values are odds ratios (OR) with 95% confidence intervals (CI). OR for SII is presented per 1000-unit increase. AUC, area under the receiver operating characteristic curve; SII, systemic immune–inflammation index.

## Data Availability

The datasets used and analyzed during the current study are available from the corresponding author on reasonable request.

## Supplementary Materials

The following supporting information can be downloaded at: https://www.mdpi.com/article/10.3390/diseases14080285/s1, Table S1: Baseline balance before and after matching; Table S2: Timing and types of thrombotic events; Table S3: Cancer-specific comparison of SII between patients with thrombosis and matched controls; Table S4: Model discrimination and calibration; Table S5: Incremental value of SII assessed by nested likelihood ratio testing; Table S6: Sensitivity analysis after additional adjustment for hemoglobin and creatinine; Table S7: Inflammatory and hematological markers in adjusted mortality models; Table S8: SII cut-off analyses for all-cause mortality; Table S9: Mortality according to oncological treatment category among thrombotic patients; Table S10: Tumor sites and antithrombotic treatment characteristics among patients with thrombosis.

## Abbreviations

The following abbreviations are used in this manuscript:

CAT	cancer-associated thrombosis
CI	confidence interval
DVT	deep vein thrombosis
ICI	immune checkpoint inhibitor
IQR	interquartile range
LMR	lymphocyte-to-monocyte ratio
NLR	neutrophil-to-lymphocyte ratio
NE	non-estimable
OR	odds ratio
PE	pulmonary embolism
PLR	platelet-to-lymphocyte ratio
ROC	receiver operating characteristic
SII	systemic immune–inflammation index
VTE	venous thromboembolism
