# Supplementary material for: Systemic Immune–Inflammation Index, Thrombotic Status, and Mortality in Patients with Cancer: A Matched Cohort Study
Source: Diseases. 2026 Aug 9;14(8):285. doi: 10.3390/diseases14080285 (PMC13512240; doi:10.3390/diseases14080285)
Supplement: Supplementary file 1 [file diseases-14-00285-s001.zip › diseases-4420225-supplementary.pdf]

Supplementary tables

Systemic immune-inflammation index and mortality in cancer-associated thrombosis

Supplementary Table S1. Baseline balance before and after matching

Variable	Thrombotic cases (n = 110)	All eligible controls before matching (n = 1910)	SMD before matching	Selected matched controls (n = 220)	SMD after matching
Age, years	68.718	64.658	0.382	69.091	−0.039
Male sex	0.527	0.535	−0.015	0.527	0.000
Cancer type, coded variable	2.609	2.816	−0.172	2.609	0.000
Coronary artery disease	0.045	0.051	−0.026	0.036	0.046
Previous myocardial infarction	0.045	0.034	0.059	0.041	0.022
Previous stroke	0.064	0.041	0.102	0.050	0.059
Diabetes mellitus	0.200	0.142	0.155	0.223	−0.056
Hypertension	0.682	0.553	0.267	0.727	−0.100
Obesity	0.245	0.244	0.003	0.282	−0.083
Atrial fibrillation	0.091	0.083	0.028	0.109	−0.061
Dyslipidemia	0.109	0.061	0.173	0.073	0.127
Vascular disease	0.036	0.026	0.060	0.055	−0.087
Valvular heart disease	0.027	0.025	0.014	0.023	0.029
Heart failure	0.136	0.051	0.296	0.123	0.041
Smoking	0.191	0.261	−0.168	0.195	−0.012
Alcohol use	0.127	0.105	0.070	0.127	0.000
Cancer stage, coded variable	2.873	2.664	0.187	2.841	0.028
Metastatic disease	0.318	0.188	0.303	0.300	0.039

For age, values represent means. For binary variables, values represent proportions. Type and Stage are shown as coded variables as provided in the Balance_summary sheet. SMD, standardized mean difference. The before-matching comparison used thrombotic cases and all eligible non-thrombotic controls from the source cohort; the after-matching comparison used the selected matched controls. Laboratory variables were not included because no before-matching SMD values for the full source cohort were provided in the Balance_summary sheet. Values are taken directly from the updated Balance_summary sheet in the matched-control file. No additional variables or values were added.

Supplementary Table S2. Timing and types of thrombotic events

Variable	Value
**Timing of thrombotic events**	
Total patients with thrombosis	110
Time-to-event data available after baseline assessment	77/110 (70.0%)
Thrombotic event before baseline assessment	33/110 (30.0%)
Time to thrombotic event, median (IQR), days	104 (30–286)
Time to thrombotic event, mean ± SD, days	237.5 ± 325.7
Time to thrombotic event, minimum–maximum, days	0–1312
Event documented on day 0	5/77 (6.5%)
Event within 7 days	13/77 (16.9%)
Event within 30 days	20/77 (26.0%)
Event within 90 days	32/77 (41.6%)
Event within 180 days	51/77 (66.2%)
Event within 365 days	60/77 (77.9%)
Event after 365 days	17/77 (22.1%)
**Types of thrombotic events**	
Deep vein thrombosis	42 (38.2)
Pulmonary embolism	47 (42.7)
Thrombosis of other sites	21 (19.1)
Total	110 (100.0)

Values are presented as median (interquartile range), mean ± standard deviation, minimum–maximum, or n/N (%), as appropriate. Percentages for event-timing categories are calculated among the 77 patients whose thrombotic event occurred at or after baseline assessment. Percentages for thrombotic event types were calculated using the total thrombotic cohort (n = 110).

Supplementary Table S3. Cancer-specific comparison of SII between patients with thrombosis and matched controls

Cancer type	Thrombotic patients, n	SII, median [IQR]	Matched controls, n	SII, median [IQR]	p value*
Respiratory	16	1384 [469–2137]	32	965 [581–2020]	0.922

Cancer type	Thrombotic patients, n	SII, median [IQR]	Matched controls, n	SII, median [IQR]	p value*
Gastrointestinal	46	792 [532–1399]	92	778 [498–1296]	0.650
Genitourinary	22	714 [490–1045]	44	660 [479–961]	0.649
Breast	17	719 [607–1088]	34	779 [453–1242]	0.849
Other	9	1481 [560–1870]	18	963 [747–1290]	0.898

*Mann–Whitney U test. Values are presented as median [interquartile range]. IQR, interquartile range; SII, systemic immune-inflammation index.

Supplementary Table S4. Model discrimination and calibration

Model	Population	Events / n	AUC	Hosmer-Lemeshow χ^2	Hosmer-Lemeshow p
Overall multivariable model	Overall matched cohort	94 / 330	0.779	7.72	0.462
Subgroup model	Thrombotic patients only	32 / 110	0.818	10.312	0.244
Subgroup model	Matched controls only	62 / 220	0.779	10.307	0.244

The overall model included SII, thrombotic status, age, sex, cancer type, stage, and metastatic disease. Subgroup models included SII, age, sex, stage, and metastatic disease. AUC, area under the receiver operating characteristic curve; SII, systemic immune-inflammation index. Cancer stage was entered as a categorical variable in all multivariable models.

Supplementary Table S5. Incremental value of SII assessed by nested likelihood ratio testing

Population / analysis	Nested model comparison	LR χ^2	df	p value
Overall matched cohort	Clinical model vs clinical model + SII	9.81	1	0.0017
Thrombotic patients only	Clinical model vs clinical model + SII	8.94	1	0.0028
Matched controls only	Clinical model vs clinical model + SII	5.11	1	0.024
Interaction analysis	Model without interaction vs model with SII × thrombotic status	3.26	1	0.071

For the overall matched cohort, the clinical model included thrombotic status, age, sex, cancer type, stage, and metastatic disease. For subgroup analyses, the clinical model included age, sex, stage, and metastatic disease. LR, likelihood ratio; SII, systemic immune-inflammation index.

Supplementary Table S6. Sensitivity analysis after additional adjustment for hemoglobin and creatinine

Model	Population	OR for SII	95% CI	p value	AUC	Hosmer-Lemeshow p	Nested LR χ^2	Nested LR p
Clinical model + SII + hemoglobin + creatinine	Overall matched cohort	1.29	1.02-1.63	0.031	0.795	0.852	21.01	<0.001

OR for SII is presented per 1000-unit increase. The model included SII, thrombotic status, age, sex, cancer type, stage, metastatic disease, hemoglobin, and creatinine. The nested likelihood ratio test compares the full model with the corresponding clinical model and therefore reflects the joint addition of SII, hemoglobin, and creatinine, not the incremental value of SII alone. The independent association of SII in this sensitivity model is represented by the OR, 95% CI, and p value for SII.

Supplementary Table S7. Inflammatory and hematological markers in adjusted mortality models

Marker	Scale	OR	95% CI	p value	Direction of association
SII	Per 1 SD increase	2.17	1.14-4.13	0.018	Higher mortality risk
NLR	Per 1 SD increase	1.62	1.08-2.43	0.021	Higher mortality risk
PLR	Per 1 SD increase	1.25	0.96-1.61	0.092	Trend toward higher risk
MLR	Per 1 SD increase	1.18	0.93-1.48	0.171	Not significant
LMR	Per 1 SD increase	0.71	0.53-0.96	0.023	Lower mortality risk
Hemoglobin	Per 1 SD increase	0.63	0.48-0.83	0.001	Lower mortality risk
Leukocytes	Per 1 SD increase	1.51	1.04-2.19	0.031	Higher mortality risk
Neutrophils	Per 1 SD increase	1.56	1.05-2.31	0.027	Higher mortality risk
Monocytes	Per 1 SD increase	1.39	1.06-1.83	0.018	Higher mortality risk

Platelets	Per 1 SD increase	1.46	1.12-1.90	0.005	Higher mortality risk

Note: Each marker was entered separately in a model adjusted for thrombotic status, age, sex, categorical stage, and metastatic disease. CI, confidence interval; LMR, lymphocyte-to-monocyte ratio; MLR, monocyte-to-lymphocyte ratio; NLR, neutrophil-to-lymphocyte ratio; OR, odds ratio; PLR, platelet-to-lymphocyte ratio; SD, standard deviation; SII, systemic immune-inflammation index.

Cancer type was not included in these exploratory marker-specific models.

Supplementary Table S8. SII cut-off analyses for all-cause mortality

Population	SII cut-off	Mortality below / at cut-off	Mortality above cut-off	p value
Thrombotic patients	SII >1000	16.9%	46.7%	0.0012
Matched controls	SII >1000	21.0%	41.6%	0.0016
Thrombotic patients	SII >1500	22.6%	50.0%	0.012
Matched controls	SII >1500	24.6%	42.2%	0.025

Cut-off analyses are exploratory and are presented to support sensitivity assessment of the relationship between elevated SII and all-cause mortality. p values were calculated using Fisher's exact test. SII, systemic immune-inflammation index.

The cut-offs were exploratory and were not prespecified; therefore, these analyses should be interpreted as descriptive and hypothesis-generating.

Supplementary Table S9. Mortality according to oncological treatment category among thrombotic patients

Treatment category	Alive, n	Dead, n	Mortality, %
No treatment	5	0	0.0
Chemotherapy	26	13	33.3
Targeted therapy	7	1	12.5
Immunotherapy	5	2	28.6
Combined therapy	21	16	43.2
Radiotherapy	14	0	0.0

Overall p value = 0.014 by Fisher-Freeman-Halton exact test for the 6 x 2 contingency table. Because several treatment categories included small cell counts or zero events, this comparison should be interpreted as descriptive and exploratory rather than confirmatory.

Legend: Treatment categories were coded as follows: 0 = no treatment, 1 = chemotherapy, 2 = targeted therapy, 3 = immunotherapy, 4 = combined therapy, and 5 = radiotherapy.

Supplementary Table S10. Tumor sites and antithrombotic treatment characteristics among patients with thrombosis

Variable	n	%
**Primary tumor site**		
Colon	24	21.8
Breast	17	15.5
Prostate	11	10.0

Larynx	10	9.1
Liver	8	7.3
Pancreas	7	6.4
Stomach	6	5.5
Kidney	5	4.5
Lung	4	3.6
Ovary	4	3.6
Skin	3	2.7
Endometrium	2	1.8
Adrenal gland	1	0.9
Brain	1	0.9
Eye	1	0.9
Hypopharynx	1	0.9
Nasal cavity	1	0.9
Parotid gland	1	0.9
Pituitary gland	1	0.9
Rectum	1	0.9
Tongue	1	0.9
**Antithrombotic treatment**		
Direct oral anticoagulant (DOAC)	50	45.5
Low-molecular-weight heparin	32	29.1
Vitamin K antagonist	9	8.2
Antiplatelet therapy	4	3.6
Missing treatment information	15	13.6

Values are presented as n (%). Percentages were calculated using the total thrombotic cohort (n = 110). Information on antithrombotic treatment was unavailable for 15 patients.
